# Prevalence of Lower Back Pain and Risk Factors in Equestrians: A Systematic Review

**DOI:** 10.3390/sports12120355

**Published:** 2024-12-19

**Authors:** Carlota Rico Duarte, Armando Raimundo, João Paulo Sousa, Orlando Fernandes, Rute Santos

**Affiliations:** 1 Escola Superior de Biociências de Elvas, Instituto Politécnico de Portalegre, 7300-110 Portalegre, Portugal; 2 CHRC—Comprehensive Health Research Centre, Universidade de Évora, 7004-516 Évora, Portugal; 3 Escola de Saúde e Desenvolvimento Humano, Universidade de Évora, 7004-516 Évora, Portugal; 4 VALORIZA—Research Centre for Endogenous Resource Valorization, Instituto Politécnico de Portalegre, 7300-555 Portalegre, Portugal

**Keywords:** equestrian athletes, equestrian sports, injury prevention, lumbar pain, musculoskeletal disorders, occupational health

## Abstract

This systematic review aimed to determine the prevalence of lower back pain (LBP) in equestrian athletes (EAs) and identify associated risk factors. Following the PRISMA guidelines, observational studies published between 2004 and 2024 in English, Portuguese, Spanish, and German were included. The review identified relevant studies through the Web of Science, EBSCO, MEDLINE, and SCOPUS (last search performed on 30 October 2024), yielding 14 studies with a total of 4527 participants. The question format for the included studies specified the population as equestrian athletes, the exposure as equestrian sports, and the outcome as lower back pain. The risk of bias was evaluated using the Observation Study Quality Evaluation tool, and six studies were deemed high-quality. LBP prevalence in EAs was higher than in the general and athlete population, with point prevalence ranging from 27.9% to 87.9%. Sport-specific factors, including workload and stable duties, were significant risk factors. Methodological inconsistencies, such as varying definitions of LBP and a lack of standardized exposure assessment, and the overall low quality of studies limited the comparability of findings. This review underscores the need for more high-quality research and tailored interventions addressing both riding and off-horse activities in EAs.

## 1. Introduction

Lower back pain (LBP) is a prevalent musculoskeletal disorder affecting the general population [1], particularly athletes [2]. While regular exercise can mitigate the risk of LBP, high levels of physical activity can paradoxically increase it [3,4]. In the context of equestrian sports (ES), back pain is frequently identified as the most common overuse injury among equestrian athletes (EAs) [5], with a notably high prevalence in this population [6].

ES are unique in that they depend on the intricate interaction between horse and rider. Historically, the scientific literature has primarily focused on the equine athlete, often overlooking the human athlete’s role [7]. However, recent research has begun to address this gap, emphasizing the physical demands placed on the rider. ES are characterized by long career spans, with athletes often beginning competitive riding as early as 6 years old [8] and continuing to compete at the highest levels, such as the Olympics, well into their 60s and 70s [9,10].

Given that previous episodes of LBP are a strong predictor of future occurrences [11], the potential impact of LBP on an EA’s career is concerning. This matter is particularly salient considering the rider’s reliance on clear and balanced physical communication with their horse [12]—a crucial aspect of performance when dealing with a 500 kg prey animal.

Over the past two decades, efforts have been made to identify the musculoskeletal complaints most affecting EAs and to understand the risk factors contributing to their development. Given the distinctive nature of equestrian sports and the high prevalence of LBP among riders, it is crucial for the equestrian community to fully understand the impacts of LBP and identify potential risk factors. However, existing studies have reported varied findings regarding the prevalence and underlying causes of LBP in this population, highlighting the complexity of establishing clear risk factors. This knowledge is essential for developing targeted, evidence-based prevention and management strategies.

The objectives of this systematic review were to determine the prevalence of LBP among EAs and identify the specific risk factors contributing to LBP in this population. By addressing these objectives, the review aimed to provide a comprehensive understanding of LBP within equestrian sports, offering informed guidance for future research on the development of effective prevention and management strategies to enhance the well-being and performance of EAs.

## 2. Materials and Methods

### 2.1. Research Design

A detailed review of observational epidemiological studies on LBP in EAs was conducted using the PRISMA statement guidelines (Preferred Reporting Items for Systematic Reviews and Meta-analysis) [13]. The only significant amendment to the original protocol was the substitution of the tool used for assessing the risk of bias and the quality of included studies with a more suitable evaluation tool, ensuring a better fit for the specific study designs analyzed. Methods of analysis and inclusion criteria were prespecified and documented in a protocol (PROSPERO database ID: CRD42024568577). The question format used for the present review was PEO: EAs constituted the population, equestrian sports were the exposure, and LBP was the main outcome.

### 2.2. Types of Studies

The studies considered for this systematic review were published in English, Portuguese, Spanish (the language in which the research team is most proficient), and German (translated for the team by a native speaker and specialist in equine sciences). The review included studies published between 1 January 2004 and 30 August 2024. Eligible studies were fully published observational studies, encompassing cohort, case–control, cross-sectional, and survey-based designs, published in scientific journals.

To qualify for inclusion, studies needed to employ descriptive and analytical observational designs that provided data on the incidence of LBP in EAs. Data collection was confined to primary sources, utilizing tools such as questionnaires, interviews, and physical assessments.

### 2.3. Types of Participants and Exposure

The review encompassed EAs: individuals of any age or sex who engage in equestrian sports, defined as activities involving horseback riding at all three gaits: walk, trot, and canter. All levels of competition and practice, ranging from leisure to professional, were included. The review considered all recognized equestrian disciplines, apart from studies focusing on driving, vaulting, para-equestrian sports, rodeo, or therapeutic use of horses, due to the specific characteristics inherent to these activities.

### 2.4. Types of Outcome Measures

The main outcome measure of the study was the prevalence of LBP among EAs, encompassing non-specific, acute, and chronic LBP (LBP characteristics such as frequency, severity, and disability were also retrieved). Additionally, the research considered secondary outcomes, including exposure data and various risk factors associated with LBP, in this population. No restrictions were placed on the definitions of LBP, methods of pain reporting, or verification.

### 2.5. Information Sources and Search

Relevant papers were identified through a comprehensive search of four electronic databases: the Web of Science, EBSCO, MEDLINE/PUBMED, and SCOPUS (last search performed on 30 October 2024). Additionally, other sources, such as reference lists of included studies, review articles, and websites (e.g., ResearchGate), were searched to obtain further relevant papers.

All search strategies are summarized in Supplementary Materials SI, Tables S1 and S2. Keywords for the database search were defined using terms related to the population and exposure combined with keywords related to outcomes of interest. These keywords were combined using “OR” and “AND” operators. When possible, restrictions were applied to search terms to include only titles, abstracts, and keywords. An example of a research phrase used in PUBMED was (“horseback rid*” OR “equestrian athlete” OR “horse rid*” OR “equitation”) AND (“Back pain” OR “Lumbar” OR “Spinal injuries” OR “Back injuries” Or “Overuse injuries”).

No filters were applied to the study design to ensure that all relevant studies were included for abstract screening. The study design was identified by analyzing full papers, looking for terms such as “epidemiology”, “retrospective”, “prospective”, “longitudinal”, “survey”, “questionnaire”, “cross-sectional”, “case–control”, and “cohort”.

### 2.6. Study Selection

To assess eligibility, two reviewers (R.S. and O.F.) with a background in equestrian sports sciences, equine sciences, health and sports sciences, and training in scientific investigation were involved in the search strategy and identification of relevant records. When discrepancies existed, they were resolved by achieving consensus. The opinion of experts in epidemiology (J.P.S.) or in the field of sports sciences (A.R.) was sought when required.

No attempt was made to rectify the reported study design. Priority was given to the design obtained from each paper title, abstract, and methods section. However, whenever it was not mentioned in the paper, the study design was defined based on the definitions given by Carlson and Morrison [14].

### 2.7. Data Collection Process

A data extraction form was developed to summarize the evidence and was pilot-tested on three randomly selected papers by one reviewer (C.R.D.). The data extraction was then verified by a second reviewer (O.F.), with any disagreements resolved through discussion. This approach enhanced the quality of data extraction and helped identify additional items to be collected. To ensure consistency and comprehensiveness, all reported characteristics of each study were considered during data extraction, even when matched with other papers.

### 2.8. Assessment of Methodological Quality and Risk Bias

The quality and risk of bias—and reporting bias—of the studies were assessed using the Observational Study Quality Evaluation (OSQE) tool [15], which has distinct versions for cohort, case–control, and cross-sectional studies, each with its own scoring system. The OSQE cross-sectional version is a subset of items from the OSQE cohort version. Specifically, the OSQE cohort and case–control versions include 14 mandatory items and 2 optional items, while the OSQE cross-sectional version comprises 7 mandatory items and 3 optional items. Higher scores indicate better study quality. Two authors (C.D. and J.P.S.) independently conducted the quality assessments of the selected studies, and consensus on the scores was achieved through meetings. A cut-off of 65% was selected, as previously reported [1,6], with studies scoring above this threshold considered to be of high quality.

For prevalence studies, the Critical Appraisal Checklist for Prevalence Studies [16] was used to assess the methodological quality and to determine the extent to which a study addressed the possibility of bias in its design, conduction, and analysis. To assess the quality of the studies, the same cut-off value of 65% was used. High-quality studies were those that obtained a score over 65%, low-quality studies scored under 65%, and any study that received a “no” for any item was not considered as a prevalence study and was excluded from the population prevalence analysis and only considered for study-specific prevalence.

### 2.9. Data Items and Analysis

When a paper lacked complete information, no assumptions were made. If supplementary materials were provided, this information was also analyzed. The review team did not contact the authors for confirmation or additional details. The primary focus was on reporting data directly available to readers. Eligible papers were coded for data extraction. The collected data items, summarized in Supplementary Materials SI, Table S3, include details on (i) study characteristics, (ii) data collection, (iii) sample details, (iv) pain details, and (v) risk factors.

Microsoft Excel [17] spreadsheets were used to organize data and for basic calculations: sums, means, and proportions. The SCALEX SP [18] calculator and Epitools [19] were used to calculate sample sizes and confidence limits for sample proportions, with the level of confidence set at 95%; these calculations were performed for studies that did not provide such information.

## 3. Results

### 3.1. Study Selection

**Figure 1:**
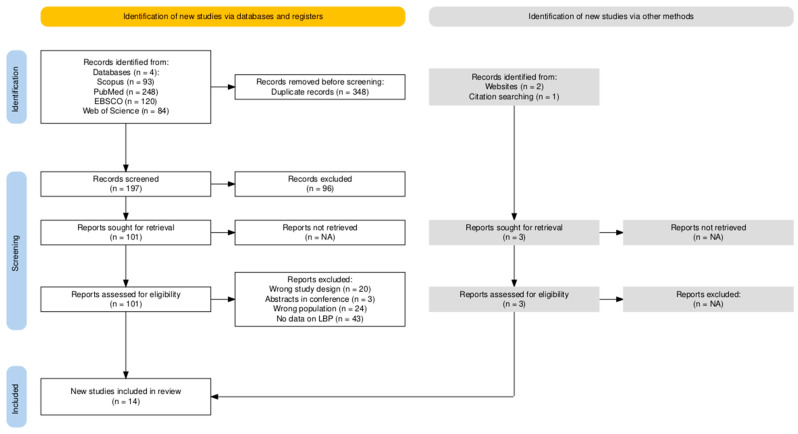
Study selection process.

A total of fourteen papers were identified for inclusion in the review. Figure 1 presents detailed information on the study selection process.

The search of the electronic databases provided a total of 545 citations. After duplication removal and language restriction, the final number of citations was 197. Of these, 96 were eliminated after screening the titles, abstracts, and keywords. One hundred and one full-text papers were examined for final confirmation of eligibility criteria. Additionally, three records were identified outside of the databases through citation tracking and relevant websites (e.g., ResearchGate). In total, 90 studies did not meet the inclusion criteria.

### 3.2. Characteristics of the Included Studies

Over the past 20 years, there has been a significant increase in the number of published observational epidemiological studies on LBP in EAs (Supplementary Materials SII, Figure S1). The design details of these studies and outcomes are summarized in Tables S4 and S5 in Supplementary Materials SII. Notably, 89.3% of the study samples originated in Europe, with a wide range of sport levels and competition statuses. However, all but one study [20] failed to clearly define rider status/skill levels. A diverse array of equestrian disciplines was represented, with only four studies [21,22,23,24] focusing on a single discipline (Table S6, Supplementary Materials SII). Across the 14 included studies, the total number of participants was 4527 (range: 19–2185). Questionnaires were the predominant data collection method (*N* = 14), with recall periods ranging from point to lifetime. Tables S6–S8 in Supplementary Materials SII provide detailed summaries of the data collection tools, procedures, recall periods, sample sizes, and participant demographics.

### 3.3. Methodological Quality

The methodological quality assessment of 14 studies is available in Supplementary Materials SII, Table S9. All studies were evaluated using the OSQE spreadsheet for cross-sectional studies, as it was the most appropriate for all study designs, despite two studies being reported as cohort studies [25,26] and one as a case–control study [20]. Only six studies achieved a score above 65%, indicating high quality (Supplementary Materials SII, Table S10). Common deficiencies across the studies included the representativeness of the sample (21.4%), assessment of the independent variable (21.4%), declaration of conflicts of interest (50%), control for confounders (57.1%), reporting of results following a protocol (100%), reporting on missing data (7.1%), analysis of effect modifiers (21.4%), and calculation of sample size (85.7%). Notably, all studies received full scores for reporting. Recall periods for pain, back pain (BP), and/LBP were only clearly stated and/or understandable in seven studies [20,25,26,27,28,29]. Although only six of the fourteen studies met the high-quality criteria, all studies were included in the review due to the limited availability of research on this topic. Study quality is addressed in the Results and Discussion sections to aid in interpreting findings.

The results of the Critical Appraisal Checklist for Prevalence Studies [16] can be found in Supplementary Materials SII, Table S11. Two studies were excluded as prevalence studies: the Lewis and Baldwin study [21] did not have an adequate sample size for precise results, and the Lewis and Kennerley study [22] did not have an appropriate sample to represent the target population. Of the remaining seven studies, three [27,28,30] were evaluated as high-quality prevalence studies and the remaining four [23,24,31,32] as low-quality prevalence studies. Sample sizes and confidence limits for sample proportions of prevalence studies can be found in Supplementary Materials SII, Table S12.

### 3.4. Demographic and Anthropometric Characteristics of the Sample

Most of the samples were dominated by female athletes (77% female average among all papers), except for four papers [21,25,27,33] (56.2% average) where the female and male samples were very even and one paper with an all-female sample [22]. In most of the studies, apart from two regarding child EAs (CEAs) [25,26] (mean age: 14.5 years) and one that included populations of all ages [30] (mean age: 33.6 years), the sample included adult EAs ranging from 18 to over 70 years of age. Seven papers did not report on the height, weight, or BMI of EAs [20,21,22,23,29,30,31]. Two papers reported on the height [28,33], weight [28,33], and BMI [33] of female and male EAs. Four papers reported on the average height, weight, and BMI of all participants [24,25,26,27]. Kraft et al. [32] only presented data on average weight and BMI with a cut-off value. Cejudo et al. [25,26] presented in both papers the body fat percentage (BF%) average value for female and male athletes.

### 3.5. Equestrian Sports

#### 3.5.1. Discipline

**Figure 2:**
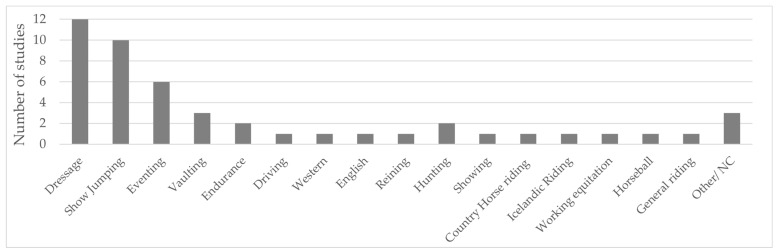
Equestrian disciplines represented in the studies and numbers of studies with populations practicing each discipline. NC—non-competitive. More details in Supplementary Materials SI, Table S6.

There are six different equestrian disciplines (EDs) recognized by the FEI and at least 50 more recognized nationally and/or internationally by different federations. The three Olympic EDs are dressage, showjumping, and eventing. The heterogeneity of EDs is visible in the selection of papers in this review, as is shown in Figure 2. The ED most represented in the papers and by the number of participants is dressage, followed by showjumping and eventing (Table 1). It is important to note that in some studies participants could report practicing more than one discipline at a time.

#### 3.5.2. Level of Sport

Comparing the level of sport was hindered by inconsistent classification systems across the studies. Some studies categorized riders by status without providing clear definitions, while others used competition levels or simply defined athletes as competitive or non-competitive, as shown in Table 2.

#### 3.5.3. Sports Practice

Measuring exposure to sports practice is crucial in these studies. For injuries, risk factors, or pain, exposure is generally quantified by the duration athletes are at risk. Understanding this workload, including the number of years spent riding and the time spent riding per week or per day, is of the utmost importance. All studies, apart from three [21,22,23], contained information on the time of equestrian sports practice (in years). One of the studies that did not provide data on years spent riding provided a statistical analysis with this variable [23]. Only eight of the studies [20,25,26,27,28,29,30,32] contained data on equestrian sports practice weekly or daily.

#### 3.5.4. Equestrian-Related Activities

Of all fourteen papers included in this review, nine [20,24,25,26,28,29,30,33] did not provide any information on daily practices in the yard and equestrian sports secondary activities (mucking out, stable-yard chores, and other activities inherent to keeping horses). Three [22,23,32] mentioned in the description of the questionnaire asking if participants needed to perform other intensive activities associated with keeping horses and what factors contributed to increased levels of pain (e.g., yard work), but they did not present any data or analysis for this variable.

### 3.6. Other Sporting Activities

Four studies [27,28,29,31] collected information regarding practices in other sporting activities and reported that 79% [31], 91% [29], and 55.9% [27] exercised or practiced other sports and that 35% [28] had a physical training program for EAs. In two studies, 34.2% of equestrians [31] and 25% of competitive showjumpers [23] used an exercise program to manage/treat pain felt.

### 3.7. Anatomic Location and Nature of Injury

**Figure 3:**
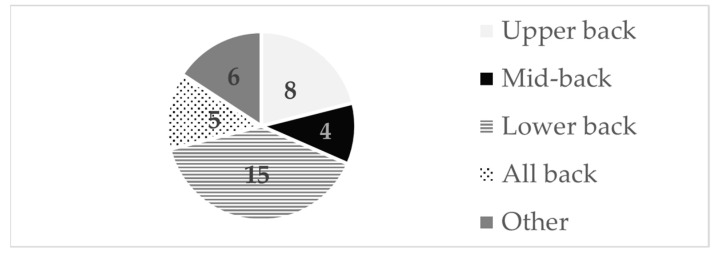
Numbers of studies reporting on pain in different trunk and body locations: upper back [20,21,23,29,30,31,33]; mid-back [20,24,29,33]; lower back [20,21,22,23,24,25,26,27,28,29,30,31,32,33]; all back [20,22,30,33]; other [21,22,23,24,31].

Pilato et al. [29] wrote a paper about injury history in collegiate EAs. They reported different types of injuries (fracture, pain/arthritis, sprain, disc injury, and others) and injuries to the spine (40.96% in the lumbar and 34.94% in the thoracic regions). A total of 6.85% of the participants suffered a fracture to the lumbar spine. Kraft et al. [32] used MRI of the lumbar spine to look for possible disc degeneration. All remaining studies [20,21,22,23,24,25,26,27,28,29,30,31,32,33] focused on pain in different bodily locations (Figure 3).

In a study about eventing riders [21], 96% of the participants reported competing with pain. Ferrante et al. [28] also reported some different musculoskeletal disorders [scoliosis, fractures, and others]. Fifty-seven percent of the riders who experienced pain in Lewis and Kennerly’s study [22] felt that pain was not associated with an old injury resulting from a fall. Three studies reported on chronic pain [22,23,31] and chronic LBP (CLBP) [28], with incidences of 83% [31], 62% [22], 67% [23], and 23.9% [28].

### 3.8. Tools and Methods for Measurement of LBP

**Figure 4:**
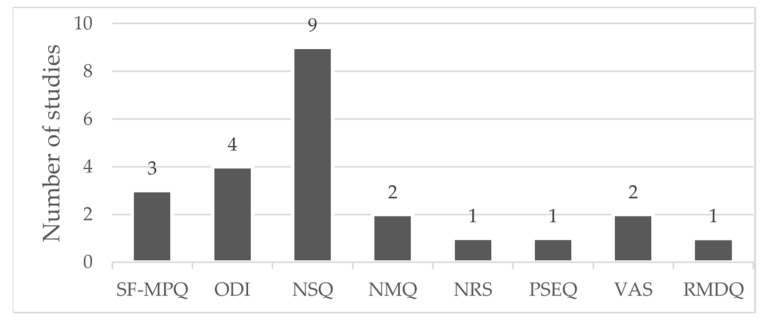
SF-MPQ—Short-Form McGill Pain Questionnaire [21,23,31]; ODI—Oswestry Disability Index (or the Oswestry Low Back Pain Disability Questionnaire) [20,31,32,33]; NSQ—non-standardized questionnaire tool [20,22,24,25,26,27,28,29,30,33]; NMQ—Nordic Musculoskeletal Questionnaire [28]; NRS—Numeric Rating Scale [28] for severity of pain; PSEQ—Pain Self-Efficacy Questionnaire [28] only for those reporting CLBP; VAS—Visual Analog Scale [30,32] to measure intensity of pain; RMDQ—Roland Morris Disability Questionnaire [27].

Tools and methods used to measure LBP (frequency, location, severity, and disability) are presented in Figure 4. The SF-MPQ is a tool used to measure the intensity of pain; it includes the present pain intensity (PPI) index and the VAS. The ODI is used to measure disability and quality-of-life impairment for adults with LBP. NMQ is used to compare lower back, neck, shoulder, and general complaints, especially musculoskeletal complaints, in epidemiological studies. NRS and VAS are used to measure pain intensity. PSEQ is used for people with chronic pain to rate self-efficacy beliefs. The RMDQ is used to evaluate LBP-related disability.

### 3.9. Lower Back Pain

**Figure 5:**
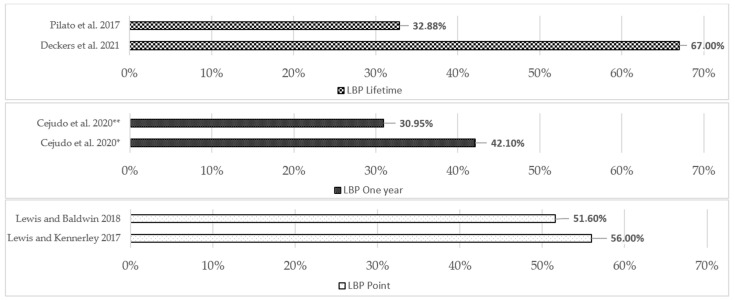
Prevalence of LBP within study population. Results of included studies divided into different recall periods. Pilato et al. 2017 [29], Deckers et al. 2021 [20], Cejudo et al. 2020 * [25], Cejudo et al. 2020 ** [26], Lewis and Baldwin 2018 [21] and Lewis and Kennerley 2017 [22].

**Figure 6:**
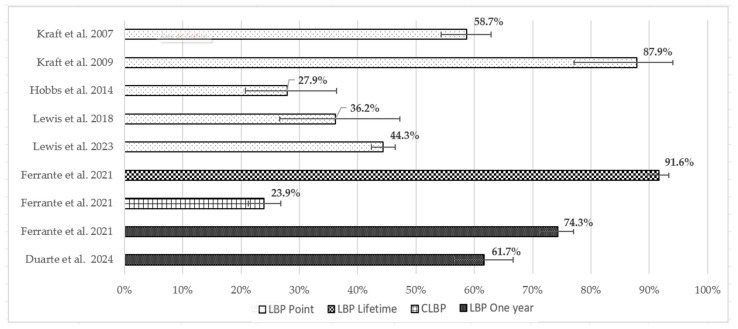
Prevalence, with confidence limits, of LBP in EAs. Results of included prevalence studies, divided into different recall periods. When not provided, confidence limits were calculated by the review team; more details are given in Table S12, Supplementary Materials SII. Kraft et al. 2007 [30], Kraft et al. 2009 [32], Hobbs et al. 2014 [24], Lewis et al. 2018 [23], Lewis et al. 2023 [31], Ferrante et al. 2021 [28] and Duarte et al. 2024 [27].

Figure 5 shows LBP prevalence within sample populations with different recall periods, and Figure 6 shows the LBP prevalence in equestrians with confidence limits.

Four studies measured LBP prevalence with a one-year recall period ranging from 61.7% to 74.3% in prevalence studies and from 30.95% to 74.3% when considering all studies. Seven studies measured LBP point prevalence; it ranged from 27.9% to 87.9% in all studies. Ferrante et al. [28] also measured LBP prevalence with a recall period of a lifetime (91.6%), 6 months (64.8%), and 1 month (46.2%) and CLBP (23.9%), defined as LBP that was present for most days in the last three months. The point prevalence within the study population of LBP in the two studies was 51.6% [21] and 56% [22].

Of all the studies, only four provided a definition of LBP. Duarte et al. [27] defined LBP as pain, discomfort, or numbness in the lower back area. Ferrante et al. [28] defined LBP as pain and discomfort localized below the costal margin and above the inferior gluteal folds, with or without referred leg pain. Cejudo et al. [25,26] gave the same definition for LBP in both of their studies: pain in the lower back that lasted for more than one week or missed training due to LBP in the previous 12 months. The period of 1 week for LBP was chosen to exclude muscle soreness. Pilato et al. [29] reported the numbers of episodes: 15.07% of the collegiate EAs had one episode, 2.74% complained of two episodes, and 15.07% had more than two episodes of pain/arthritis in the lumbar spine. Hobbs et al. [24] categorized participants with lumbar pain by posture type, and the most frequent posture types of participants with LBP were normal, kyphotic/lordotic, and swayback. In Kraft et al.’s study [32], the EAs had a significantly higher intensity of LBP than the controls; the prevalence of LBP in the control group was 33%.

### 3.10. Duration and Frequency of Symptoms

Only five studies published information on the duration and/or frequency of symptoms. Fifty-four percent of the participants in the Lewis et al. study [31] experienced pain (regardless of location or intensity) for over 6 years. A study on LBP in Italian EAs [28] reported that participants who experienced LBP during their lifetime had an average of 15 episodes, and participants with LBP in the last year had an average of 5 episodes. The average length of episodes (regardless of time prevalence) was 3 days. Pilato et al. [29] divided injury frequency into one, two, or more than two episodes and presented data on spine and pelvis injury frequency; the type of injury with a higher number of responses of two or more episodes was pain/arthritis located in the thoracic spine, followed by the lumbar spine and the cervical spine. The median LBP duration in a study of competitive showjumpers [23] was 2 to 3 years. Kraft et al. [30] reported data on the frequency of participants’ BP: 59.3% had BP occasionally, 25.2% had BP daily, and 15.6% never had BP.

### 3.11. Consequences of Pain

The main consequence of pain [21,22,23,31], LBP [27], and CLBP [28] is limitation in performance whilst riding (72.7% [31], *p* < 0.05 [28], 85% [23], 63.1% [27]) or competing (55% [21], 59% [22]). Lewis and Kennerly [22] found a statistically significant association between experiencing pain and perceptions of pain negatively affecting performance. Riders’ perceptions of how pain affected their performance were reported in four studies [21,22,23,31]. Common effects felt by EAs are postural asymmetry, limited and reduced ROM, irritability, earlier onset of fatigue, lack of concentration, and anxiety. A study on Italian EAs [28] found that CLBP was associated with time lost in the sport (*p* < 0.001), medication consumption (*p* < 0.001), and restriction of participation (*p* < 0.001). Hobbs et al. [24] stated that pain avoidance during riding could increase the prevalence of postural defects and muscle imbalances in higher-level riders. Furthermore, Cejudo et al.’s [25] results suggest that LBP impacts trunk proprioception and stability in CEAs.

#### 3.11.1. Levels of Pain, Severity, and Levels of Disability

**Figure 7:**
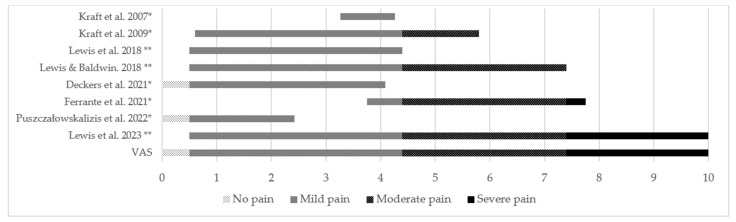
Levels of pain felt by equestrians based on VAS ratings [34]. Location of pain: LBP [23,28,31,32,33]; BP [20,30]; pain in general [21]. * Values of intensity of pain given as quantitative variables (medians with standard deviations above and below). ** Values of intensity of pain given as qualitative variables. Kraft et al. 2007 [30], Kraft et al. 2009 [32], Lewis et al. 2018 [23], Lewis and Baldwin 2018 [21], Deckers et al. 2021 [20], Ferrante et al. 2021 [28], Puszczałowskalizis et al. 2022 [33] and Lewis et al. 2023 [31].

Eight studies reported on the level of pain experienced by participants based on results of the VAS [20,21,23,30,31,32], NRS [28], and ODI [33] (question one of the ODI questionnaire). Levels of pain experienced are represented in Figure 7. Most equestrians in these studies felt mild or moderate levels of pain in general, as well as LBP and mild BP. Kraft et al. [32] found significant differences in the intensity of LBP between riders and controls. Deckers et al.’s [20] and Ferrante et al.’s [28] pain intensity levels were for all athletes with pain in all recall periods—lifetime, one year, one month, and chronic. The remaining papers measured pain levels for present LBP.

Five studies measured disability caused by BP [20], LBP [27,28,32,33], and CLBP [28]. A total of 26.4% of the respondents in the Ferrante et al. [28] survey had a disability in daily living activities, and athletes with CLBP had higher values of disability than those with LBP. These authors did not find any correlations between the severity of pain and self-efficacy in participants with LBP. Levels of disability ranged from no disability [20,32,33] and minimal disability [20,32,33] to moderate disability [33]. Duarte et al. [27] used a cut-off value to determine functionality or dysfunctionality in RMDQ results. Of the participants with LBP, 49.5% had dysfunctionality; nevertheless, the RMDQ mean score was 5.39—higher than the cut-off value of ≥4 for dysfunctionality. Two [23,31] studies mentioned in their methodology using the ODI to measure the impact of pain on equestrians’ general life and well-being, but the results of the ODI could not be found by the review team.

#### 3.11.2. Time Loss

In Lewis et al.’s [31] survey, a total of 42% of participants reported that pain or injury had stopped them from riding at some point in life. Time off riding due to pain ranged from a few days to 15 years and even prevented some from returning to riding permanently. Another survey [28] concluded that 28.5% of EAs with LBP or CLBP had suspended sporting activities and that athletes with CLBP suspended sporting activities more frequently. In a study on competitive showjumpers [23], 15% reported that pain had prevented them from riding; time off ranged from one day periodically to one year.

#### 3.11.3. Pain Management Techniques

The pain management techniques reported in studies were medication, consultation with a physician, and various types of therapies (e.g., physical therapy, therapy, osteopathy, and massage). The percentages of equestrians with pain who used medication were 75% [31], 96% [21], and 37.2% [28]. The percentages of EAs who used over-the-counter medication were 51.1% [31], 93% [21], 67% [23], and 51.4% [22]. The percentages of EAs who used medication with a medical prescription were 23.9% [31], 3% [21], 9% [23], and 16.2% [22]. In a study on collegiate EAs [29], 16.44% regularly used pain medication. Among the equestrians who had pain and sought treatment to help manage it, 33% [33], 36.7% [28], and 49.6% [30] had visited a physician. The most common therapies used by equestrians with pain were physical therapy (47.7% [31], 38.5% [33], 61.69% [28], 19% [21], 47% [23], and 18.9% [22]) and massage (12% [33] and 29% [23]).

### 3.12. Risk Factors, Associations, and Contributing Factors for LBP

Tables S13–S16 (Supplementary Materials SIII) report data on risk factors, associations, and contributing factors for pain, BP, and LBP. Variables with statistically significant associations were classified as “risk factors”, those without significant associations were classified as “not risk factors”, while those without significant statistical analysis were considered “contributing factors” or “not contributing factors”. The data are categorized into population characteristics (Tables S13 and S15) and exposure characteristics (Tables S14 and S16). Due to the variability in data analysis, population characteristics, and reporting methods, it was not possible to combine findings for most variables.

Two studies [27,28] found that sex was not a risk factor for the one-year prevalence of LBP [27], LBP incidence over a lifetime or for a one-year period [28], CLBP incidence [28], or LBP-related disability and functionality issues [27]. However, Puszczałowska-Lizis et al. [33] reported that women had a higher risk of experiencing pain in the lumbar back pain compared to men. Regarding anthropometric characteristics, height [25,26,28], weight [25,26,28], and BMI [25,26,27,28,32] were not identified as risk or contributing factors for one-year LBP incidence [25,26,27,28], CLBP [28], dysfunctionality due to LBP [27], or disc degeneration disease [32]. However, Duarte et al. [27] observed that higher BMI scores were significantly correlated with increased disability scores. Ferrante et al. [28] identified weight as a substantial risk factor for lifetime LBP prevalence. Only two studies [25,26] investigated BF% and yielded opposing conclusions, despite having similar populations and methodologies. One study found no correlation between BF% and LBP [26], while the other identified BF% as a prominent risk factor for LBP in CEAs, with a cut-off value of BF% > 23% [25]. Two high-quality studies provided somewhat contradictory results concerning age as a risk factor. Ferrante et al. [28] found that younger age was a risk factor for LBP for both lifetime and one-year incidence. In contrast, Duarte et al. [27] found that older age was a risk factor for LBP-related dysfunctionality. No significant associations were found between age and CLBP [28], disability scores [27], LBP in CEAs [25,26], or one-year LBP incidence [27]. Kraft et al. [32], using magnetic resonance imaging (MRI), concluded that incipient disc degeneration was not a risk factor for LBP point prevalence and found no relationship between trunk/leg-length coefficient and disc degeneration disease. Engaging in sports other than equestrian activities did not pose a risk factor for lifetime [28] or one-year [27,28] LBP incidence, nor for LBP-related disability [27]. Two studies on CEA populations [25,26] which had similar characteristics found significant asymmetries in range of motion (ROM) (more information in Table S13, Supplementary Materials SIII] and trunk muscle endurance (ISBE) [25] between dominant and non-dominant limbs in all participants, regardless of LBP incidence. Nonetheless, these studies determined that higher values in ROM (hip total rotation) [25] and lower values in ROM (hip adduction with hip flexed (HAD-HF), flexion of knee (KF) [26], and lower-trunk muscle endurance (isometric side bridge endurance (ISBE) and ISBE in non-dominant side)) [25] were risk factors for LBP incidence, with cut-off values of HAD-HF ≤ 26°, KF ≤ 128°, and ISBE ≤ 65 s.

Practicing equestrian sports professionally, rather than as a hobby, was identified as a strong risk factor for LBP incidence, disability, and dysfunctionality caused by LBP [27]. Additionally, 43% of the equestrian population [27] considered riding a contributing factor to the intensity of pain experienced. In contrast, Kraft et al. [32] found that being an equestrian athlete did not pose a risk for T2-weighted signal alterations of the lumbar spine (disc degeneration), and Duarte et al. [27] did not find a significant correlation between LBP prevalence and the level of equestrian sports practiced, whether professionally or as a hobby. Equestrian discipline was not a risk or contributing factor for LBP prevalence [27], intensity, disability, disc degeneration disease [32], or CLBP incidence [28]. However, Kraft et al. [32] noted that practicing dressage might contribute to T2-weighted signal alterations in the lumbar spine. Ferrante et al. [28] found a significant relationship between equestrian discipline and lifetime LBP prevalence, but this result should be interpreted cautiously due to the small sample sizes in some disciplines and discipline characteristics. No correlations were found between the level of riding (as indicated by sport license) and LBP or CLBP incidence [28].

Workload was a significant risk factor for LBP [27] and CLBP [28] incidence when it reached 5 to 6 h/week [28], exceeded 7 h/week [27], or surpassed 13 h/week [28]. Other studies did not find correlations between workload and point [32], lifetime [28], or one-year LBP incidence [25,26,28]; LBP intensity [32]; or LBP-related disability [27]. The duration of equestrian sports practice (in years) was not identified as a risk or contributing factor for LBP incidence [27,28] or LBP-related disability [27].

One high-quality study found that performing stable duties was a major risk factor for LBP incidence, though it did not affect functionality [27]. Specifically, stable duties like mucking out appeared to be contributing factors to higher disability scores and LBP intensity. Grooming activities and lunging horses also contributed to LBP intensity in 27% and 26% of equestrians [27].

## 4. Discussion

This systematic review aimed to clarify the prevalence and risk factors associated with LBP in equestrian athletes, as this population is uniquely exposed to physical demands distinct from those in other sports. Equestrian sports combine high-intensity activities with repetitive motion and prolonged postures, placing specific biomechanical stresses on the lower back. Given these unique demands, understanding the prevalence of LBP in equestrians compared to the general and athletic populations provides insight into the potential need for targeted interventions. The findings of the present review indicate that the prevalence of LBP among equestrians is higher than in the general population [1] across all recall periods—lifetime, one-year, and point. The lifetime prevalence of LBP in EAs was measured in only one high-quality study [28] and was found to be higher than the pooled prevalence for athletes [2,6]. One-year LBP prevalence in equestrians, reported by all prevalence studies, is higher than the pooled one-year prevalence in athletes [2,6] yet lower than the prevalence range for horse-riding athletes reported by Wilson et al. [6]. Regarding point prevalence of LBP in equestrians, based on both high- and low-quality studies, it is generally higher than the pooled point prevalence in athletes [2,6], except for one low-quality study [24] where the point prevalence was lower than the pooled values in athletes [6]. Given this, although the prevalence of LBP is high in athlete populations—particularly since athletes are less likely to have comorbidities compared to the general population [6]—it generally appears to be even higher among equestrians. Similarly, this pertains to CLBP prevalence being higher in EAs [28] in comparison to the general population [1] with different physical activity levels—low, moderate, and high [35]. Additionally, incidence could not be established, since studies did not report a minimum symptom period or whether LBP episodes were recurrent or not. In the present review, 57.1% of the studies—high- and low-quality—used validated tools or at least clear definitions to identify LBP. Furthermore, as only 28.6% of the studies provided a definition of LBP, attention must be given to the definition of BP since variations in definitions can result in different prevalence estimates [2]. Wilson et al. [6] highlighted the urgency of creating a definition of LBP for athletes for use in research. Additionally, in the present review, the team noted that the terms BP and LBP were used interchangeably at times. The same was noted in other reviews [6].

It has been determined that a prominent risk factor for LBP is a previous LBP episode [6], that is, a history of LBP. The present review’s findings cannot support this conclusion; only 35.7% of studies published data on the duration or frequency of symptoms, and these variables were not comparable due to methodological heterogeneity. The reported levels of pain in equestrians ranged from none to severe, yet most pain was mild or moderate—a finding in line with adolescent athlete [36], elite athlete [37,38], and non-athlete [37] populations with LBP. Research has proven that intensity and disability caused by LBP are correlated [39]. The most common levels of disability caused by LBP in equestrians were no disability and minimal disability, which seems to be similar to the athlete population [38]. On the other hand, EAs [27] seem to be more prone to dysfunctionality than elite athletes [38]. The disability results could be lower than expected due to the lack of sensitivity of the tools used in the assessment of disability in athletes—in their sports and exercise activities [40]—athletes could have limitations to their athletic performance and yet have little or no disability in their daily activities [38]. A systematic review of instruments used to assess BP in athletes [41] published in 2023 suggested that future research on BP in athletes should use the Athlete Disability Index [38].

The results of the present review show that equestrians tend to use medication to manage pain more than other non-pharmacological therapies. Pain is commonly self-managed by athletes using over-the-counter pain medications or supplements, suggesting that information specifically aimed at athletes on the safe and efficacious use of pain medications is necessary [42]. Managing pain in elite athletes must balance the tension between ignoring or masking pain and recognizing its protective role in the presence of injury [42]. The mission of the World Anti-Doping Agency is to promote clean sport, and to support this goal, understanding the prevalence of LBP among equestrians is crucial. This knowledge can help evaluate treatment strategies to ensure that EAs have access to therapists and other pain management methods, reducing the reliance on self-medication [22].

Living, training, and competing in pain can carry significant consequences. Most EAs in pain—generally, in the back or in the lower back—feel limitations in their performance when riding and competing. The literature has shown that LBP and BP reduce athletic performance in training and competition [43,44,45,46]. Moreover, performance is not limited to sports. A study characterizing injuries suffered by mounted and non-mounted police officers [47] concluded that the most common injuries in mounted police officers were to the lower back and musculoskeletal in nature. Given their responsibility to protect the public, a decline in police officers’ performance could lead to serious injury or even death for themselves, their fellow officers, or members of the community they serve [Orr et al. 2017 and Simas et al. 2022, cited in [47]]. Other consequences of LBP are effects on participation [6] in training and competition, the high costs of treatment, decreased quality of life [46] and functional impairment [6]. Furthermore, it is known that asymmetry has an impact on equestrian performance [12]. Significant asymmetries of ROM and ISBE have been detected in EAs [25], and pain avoidance in riding can increase the prevalence of asymmetry [24]. Further research focusing on LBP and asymmetry in EAs is needed to help understand if asymmetry is a consequence of pain or if pain is a consequence of underlying asymmetries. A systematic review and meta-analysis on postural asymmetries and LBP concluded that lumbopelvic mechanisms may be altered in individuals with LBP, yet no definitive conclusions could be drawn [48].

As in the present review, results regarding the risk anthropometric parameters pose to LBP tend to be inconsistent and inconclusive, especially in the athletic population. In the general population, LBP can be experienced at any age, but prevalence and incidence are higher in older individuals [49]. However, Shiri et al. [50] found that LBP slightly declined with increasing age, while lumbar radicular pain increased with age. In sports, the evidence was insufficient and inconsistent, making it impossible to establish any association between age and LBP [2,6,51]. The same seems to be true for sex. In the general population, LBP and lumbar radicular pain affect more women [49,50], yet in sports the evidence is inconsistent [2,6,51]. In the present review, there is strong evidence indicating that height is not a risk factor for LBP in the general population [50] and athletes [51]. Weight, BMI, and BF% seem to be consistent risk factors for LBP across the literature [6,50,51,52]. In EAs, there was inconsistent evidence to demonstrate an increased risk. Other associations, such as the practice of other sporting activities (different from the main sport) and disc degeneration in athletes and EAs, were also inconsistent [6,51,53]. Altered lumbar ROM—flexion and extension—have been considered strong risk factors for LBP [51]. In the present review, altered ROM was considered a predictive factor for LBP in CEAs, yet it is not possible to compare the relevant findings due to assessment heterogeneity.

Considering all this information, it can be assumed that the higher prevalence of LBP in equestrians is more closely related to sport-specific variables than to the anthropometric characteristics of the riders. In the present work, there is strong evidence that the type of equestrian discipline does not significantly impact LBP. While disciplines differ in nature and biomechanical demands on both horse and rider, the daily work of the equine and equestrian athlete is similar across them. Training sessions often overlap, sharing common characteristics, and the widely accepted correct rider position remains consistent across all disciplines, varying only with specific training or tasks. Competition level, skill level, years of sport, and workload are exposure variables that correlate—an athlete at a higher skill and competition level naturally has more experience coming from more years and a higher workload in the sport. Although there is strong evidence that years of exposure to sport and high volumes of training are risk factors for LBP prevalence [6], other authors could not find evidence for these associations [2,51]. This inconsistency is also reflected in the present review, where findings for these variables—competition level, skill level, years of sport, and workload—were inconsistent. However, this may be attributed to poor assessment stemming from the lack of standardized tools for measuring exposure in equestrian sports. Future research on EAs should focus on developing and validating survey tools specifically designed for this population.

Horse riding is one of the sports with the highest prevalence of LBP in elite athletes [6], implying that the functional characteristics of equestrian sports may be a key factor in the high prevalence of LBP. Horse riding appears to generate whole-body vibrations [54], which in turn increases the risk of LBP [55]. Moreover, the present review indicated that activities related to the maintenance and management of the equine partner appear to increase the risk of LBP. The literature has found that bent and twisted back positions—common in some of these activities—create harmful stress loads [56] and increase the risk of musculoskeletal problems [57]. Additionally, heavy workloads, repeated lifting, and the accumulation of stress from flexed, rotated, and awkward lumbar spine positions were identified as moderate to strong risk factors for LBP [58]. Future research should make a concerted effort to include, rather than overlook, the off-horse workloads inherent in equestrian sports.

No definitive risk factors for LBP in EAs have been identified yet, highlighting the need for further scientific research on this topic. To advance our understanding, it is crucial to focus on the following areas:

- Study Quality: Conducting higher-quality studies is essential to provide more substantial evidence regarding which variables pose risk factors for LBP and which do not.
- Research Tools: There is a pressing need to develop standardized questionnaires that address key questions, enabling researchers to better understand the prevalence of LBP in EAs and the factors contributing to its existence.

Improving these aspects will help clarify the underlying causes of LBP in this population and inform more effective prevention and treatment strategies.

## 5. Limitations

This systematic review has several limitations that should be acknowledged. First, the review process was not blinded, which could have introduced bias, as the reviewers were aware of the study authors and their affiliations. This issue is particularly pertinent given that one of the included studies shares the same main author as this review. To minimize potential bias, the quality assessment for all studies, including this one, was also conducted by a reviewer not involved with the article in question. Another limitation lies in the tools used to assess study quality and prevalence, which were originally developed for medical and health studies. These tools may lack the sensitivity required to accurately evaluate research specific to athlete populations, potentially affecting the reliability of the quality assessments. Additionally, the interchangeable use of the terms “back pain” and “lower back pain” in some studies complicated data interpretation, as these terms were sometimes conflated. During the full-text screening and data extraction, judgments had to be made regarding whether the studies specifically addressed LBP, introducing a degree of subjectivity. Furthermore, there was a challenge with the definition of “point prevalence”, as several studies did not clearly report the specific time window in which athletes were asked about their pain. In many instances, the review team had to infer that the reported prevalence referred to point prevalence based on the context, but this was not explicitly stated. This assumption may have led to inconsistencies in the reported prevalence estimates. Lastly, the review was limited to peer-reviewed articles, excluding other sources like abstracts, reports, and theses. A notable limitation in the present review was the challenge of accurately assessing exposure to risk factors due to the absence of standardized tools specific to equestrian sports. Proper exposure assessment is crucial for understanding injury and illness risk, yet current tools are generally designed for other sports contexts and may not capture the unique demands of equestrian activities. In equestrian sports, where training often includes holistic routines beyond discipline-specific sessions, exposure factors like hours spent riding, type of horse, and regular stable management tasks (e.g., grooming and mucking out) are critical but inconsistently recorded. This lack of standardized, equestrian-specific exposure measures likely influenced the precision of risk estimates across studies, limiting the comparability of findings and the review’s ability to quantify risk factors effectively. Developing tailored tools for equestrian contexts is essential to advance accuracy in future research and foster evidence-based prevention strategies in the field. A further limitation encountered was the methodological heterogeneity across studies, particularly concerning rider status and skill level. These variables were challenging to categorize consistently, as competition level alone does not fully capture rider expertise. The absence of standardized, clear definitions meant that the skill levels and competitive statuses of equestrian athletes could not be uniformly assessed. Future research would benefit from clearer definitions regarding skill level, affiliation status, and competition specifics to improve comparability and ensure that samples accurately represent different experience levels. Despite these limitations, the review provides valuable insights into the prevalence of LBP in EAs and highlights the necessity for further, more precise research in this area. Future studies should prioritize the development and validation of sport-specific tools for assessing LBP risk factors in equestrian athletes. By focusing on sport-specific variables and improving research quality, the equestrian community can better understand and mitigate LBP risks, ultimately enhancing athlete well-being and performance.

## 6. Conclusions

This systematic review underscores the heightened prevalence of LBP among equestrian athletes compared to the general population and other athletic groups. While some evidence points to sport-specific factors, such as the physical demands of riding and associated tasks, as potential contributors to this increased prevalence, definitive risk factors remain elusive due to methodological inconsistencies and a lack of standardized assessment tools. The findings highlight the need for higher-quality research focused on the unique characteristics of equestrian sports.

## Figures and Tables

**Table 1:** Numbers and percentages of papers and participants represented in each equestrian Olympic discipline.

	Papers (*n*, %)	Participants (*n*, %)
Dressage	12, 85.7	2310, 51
Showjumping	10, 71.4	1996, 44.1
Eventing	6, 42.9	644, 14.2

**Table 2:** Rider status, level of competition, and competition status of participants in each study.

	Rider Status	Level of Competition	Competitive/Non-Competitive
Deckers et al. [20]	Professional and Amateur	National	Competitive
Lewis and Baldwin. [21]	-	International	Competitive
Lewis and Kennerley. [22]	Elite	International	Competitive
Lewis, Dumbell, and Magnoni. [23]	Recreational, Amateur, and Professional		Competitive
Hobbs et al. [24]	-	-	Competitive
Kraft et al. [32]	Elite	National/International/Olympic	Competitive
Duarte et al. [27]	Hobby and Professional	-	-
Ferrante et al. [28]	-	Sport license *	Competitive/non-competitive
Cejudo et al. [25]	-	-	Competitive
Cejudo et al. [26]	-	-	Competitive
Pilato et al. [29]	-	Intercollegiate	Competitive
Lewis et al. [31]	Leisure, Amateur, and Professional	-	Competitive/non-competitive
Puszczałowska-lizis et al. [33]	Amateur	-	-
Kraft et al. [30]	-	Performance classes **	Competitive

* As defined by the Italian National Equestrian Federation. ** As defined by the German equestrian federation.

## Data Availability

The raw data supporting the conclusions of this article will be made available by the authors upon request.

## Supplementary Materials

The following supporting information can be downloaded at: https://www.mdpi.com/article/10.3390/sports12120355/s1, Supplementary Materials SI—Table S1: Search strategy performed in all databases; number of articles found in each search; Table S2: Key words selected regarding population and exposure and outcome of interest; Table S3: Summary of data items collected from included studies; Table S4: Study design features (*n* = 14); Table S5: Study outcomes (*n* = 14); Table S6: Sample details of included studies; Table S7: Data collection tools, dissemination procedure, and sample size with details; Table S8: Detailed data collection tools of included studies; Figure S1: Number of publications per period. Supplementary Materials SII—Table S9: Detailed information on OSQE tool—cross-sectional studies, with comments and explanation; Table S10: Quality score of all included studies and quality taxonomy; Table S11: JBI critical appraisal checklist for included studies reporting prevalence data; Table S12: Confidence intervals of LBP prevalence. Supplementary Materials SIII—Table S13: Population characteristics (demographic and anthropometric) that do not pose a risk or do not contribute to pain; Table S14: Exposure characteristics (related to equestrianism) that do not pose a risk or do not contribute to pain; Table S15: Population characteristics (demographic and anthropometric) that pose a risk or contribute to pain; Table S16: Exposure characteristics (related to equestrianism) that pose a risk or contribute to pain.
