# Supplementary material for: Prevalence of Lower Back Pain and Risk Factors in Equestrians: A Systematic Review
Source: Sports (Basel). 2024 Dec 19;12(12):355. doi: 10.3390/sports12120355 (PMC11679230; doi:10.3390/sports12120355)
Supplement: Supplementary file 1 [file sports-12-00355-s001.zip › Supplementary Materials SI.pdf]

Supplementary Materials SI

1. Search strategy

Table S1. Search strategy performed in all databases, number of articles found in each search.

Database	Filters /restrictions	Nº of articles
**Scopus**	AT, A, K	134
	AT, A, K (2004-PRESENT)	96
	AT, A, K, English, Portuguese and Spanish (2004-PRESENT)	80
	AT, A, K, German (2004-PRESENT)	13
	**Total**	**93**
**PubMed**	All PE words & Lower back pain	33
	All PE words & Lower back pain (2004 – Present)	30
	All PE words & Lower back pain (2004 – Present), E,P&S G	27
	All PE words & Lumbago	22
	All PE words & Lumbago (2004 – Present)	20
	All PE words & Lumbago (2004 – Present), E,P&S G	18
	All PE words & lumbar pain	33
	All PE words & lumbar pain (2004 – Present)	28
	All PE words & lumbar pain (2004 – Present), E,P&S G	25
	All PE words & dorsalgia	72
	All PE words & dorsalgia (2004 – Present)	67
	All PE words & dorsalgia (2004 – Present), E,P&S G	62
	All PE words &lower spine pain	7
	All PE words &lower spine pain (2004 – Present)	6
	All PE words &lower spine pain (2004 – Present), E,P&S G	5
	All PE words & spinal injur*	82
	All PE words & spinal injur*(2004 – Present)	58
	All PE words & spinal injur*(2004 – Present), E,P&S G	49
	All PE words & back pain	72
	All PE words & back pain (2004 – Present)	67
	All PE words & back pain (2004 – Present), E,P&S G	62
	**Total**	**248**
**EBSCO**	Txt	246
	Txt, 2004-2024	177
	Txt, English and Spanish, 2004-2024	153
	Txt, English and Spanish, 2004-2024, academic journals	112
	Txt, German, 2004-2024, academic journals	8
	**Total**	**120**
**Web of science**	Txt	97
	Txt, 2004-2024	84
	Txt, English, Portuguese, Spanish and German, 2004-2024	84
	**Total**	**84**

AT – Article title; A – Abstract; K – keywords; PE – Population and exposure keywords; E, P & S – language restrictions; G – German Txt – Full text;

Table S2. Key words selected regarding population & exposure, and outcome of interest

Population and exposure	Outcomes	Population and exposure (contin.)	Outcomes (contin.)
Horseback rider	Low back pain	Equestrian	Spinal injuries
Horseback riding	Lower back pain	Dressage	Back injuries
Equestrian athlete	Back pain	Eventing	Overuse injuries
Horse riding	Lumbar back pain	Showjumping	
Horse rider	Lumbar pain		
Equitation	Lumbar spine		

These keywords were combined using "OR" and "AND" operators to facilitate search (e.g. ["Equestrian" OR "Horse rider"] AND ["Lower back pain" OR "Overuse Injuries"])

2. Study details – Tables and content

Table S3. Summary of data items collected from included studies

Categories	Items
**Study characteristics**	(1) Year of publication; (2) Study design; (3) Outcomes; (5) Statistical analysis;
**Data collection**	(1) Sources; (2) Country; (3) Tools and methods; (4) Injury categorization; (5) Riding discipline;
**Sample details**	(1) Sex; (2) Age; (3) Height; (4) Weight; (5) BMI; (6) Body fat percentage; (7) Riding level; (8) Competition level; (9) Skill level; (10) Time practicing sport; (11) Workload; (12) Equestrian related activities;
**Pain details**	(1) Anatomical location; (2) Nature; (3) Prevalence; (4) Incidence; (5) Number of occurrences; (6) Level of pain; (7) Pain management; (8) Time loss; (9) Level of disability.
**Risk factors**	(1) Risk factors; (2) Not risk factors; (3) Contributing factors; (4) Not contributing factors;

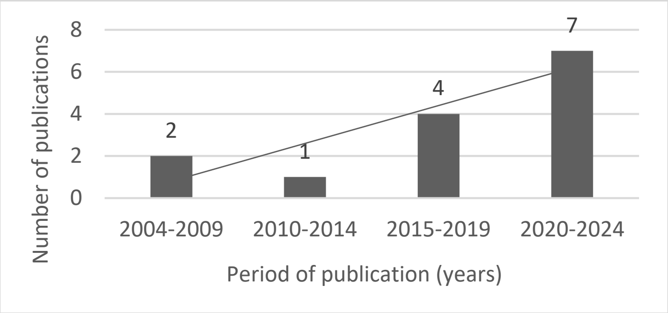

Figure S1. Number of publications per period

Table S4. Study design features (n=14)

Design	Statistical analysis	References
**Cross-sectional**	Descriptive	*Lewis et al. [31]<br>*Pilato et al. [29]
	Analytical	Duarte et al. [27]<br>*Puszczałowska-Lizis et al. [33]<br>Ferrante et al. [28]<br>*Lewis, Dumbell & Magnoni. [23]<br>*Lewis & Baldwin. [21]<br>*Lewis & Kennerley. [22]<br>*Hobbs et al. [24]<br>Kraft et al. [32]<br>*Kraft et al. [30]
**Cohort**	Analytical	Cejudo et al. [25]<br>Cejudo et al. [26]
**Case control**	Analytical	Deckers et al. [20]

*Papers that did specify study design

Table S5. Study outcomes (n=14)

Outcomes	N	References
**Low back pain**	5	Duarte et al. [27]; Ferrante et al. [28]; Cejudo et al. [25]; Cejudo et al. [26]; Kraft et al. [32];
**Back pain**	3	Puszczałowska-lizis et al. [33]; Deckers et al. [20]; Kraft et al. [30];

**Equestrian related injury**	1	Pilato et al. [29];
**Pain**	4	Lewis et al. [31]; Lewis & Baldwin [21]; Lewis, Dumbell & Magnoni [23]; Lewis & Kennerley [22];
**Posture**	1	Hobbs et al. [24];

Table S6. Sample details of included studies

Reference	Country	Source (n)	Sample	Equestrian discipline	Age group
**Duarte et al. [27]**	PT	Equestrians of the Portuguese equestrian federation	-	Dressage<br>Show Jumping<br>General riding<br>Eventing<br>Endurance<br>Horseball<br>Working equitation	Adults
**Lewis et al. [31]**	GB	Equestrian population of UK	Leisure, amateur & professional	Dressage<br>Show jumping<br>Eventing<br>Hunting<br>Showing	Over 35 y.o.
**Puszczałowska-lizis et al. [33]**	PL	Equestrian centers	Amateur	Dressage<br>Show jumping<br>Hacking	40-45
**Ferrante et al. [28]**	IT	Members of Italian national equestrian federation	Competitive<br>Non-competitive	Dressage<br>Show jumping<br>Eventing<br>Country horse riding<br>Reining<br>Endurance<br>Vaulting<br>Driving<br>Other	Adults
**Deckers et al. [20]**	BE	Equestrian population of Belgium	Professional<br>National competition<br>Competitive level	Dressage<br>Show jumping<br>Eventing<br>Icelandic riding	18-60
**Cejudo et al. [25]**	ES	Murcia regional team	Competitive	Dressage<br>Show jumping	12-17
**Cejudo et al. [26]**	ES	Equestrian technical camps	Competitive	Dressage<br>Show jumping	9-18
**Lewis & Baldwin. [21]**	GB	Hartpury international horse trials	International (1* to 3*)	Eventing	18-55
**Lewis, Dumbell & Magnoni. [23]**	GB	Equestrians of the United Kingdom	Competitive<br>Professional<br>Amateur<br>Recreational	Show Jumping	Adults
**Pilato et al. [29]**	US	Intercollegiate equestrian team	Intercollegiate competitive level	English<br>Western<br>Eventing<br>Hunt<br>Dressage	Adults

**Lewis & Kennerley [22]**	GB	Hartpury festival of dressage	International (3*)	Dressage	19-52
**Hobbs et al. [24]**	GB & US	British dressage camp & Michigan state university	Competitive	Dressage	Adults
**Kraft et al. [32]**	DE	National training camps	Elite	Dressage<br>Show jumping<br>Vaulting	18-41
**Kraft et al. [30]**	DE	Rhineland Equestrian sports association	Performance classes	Dressage<br>Show jumping<br>Vaulting	All

Table S7. Data collection tools, dissemination procedure and sample size with details

Reference	Tools (timeframe)	Recall period	Procedure	Participants (number, sex)
**Duarte et al. [27]**	Quest. (retrospective career)	One-year	Indirect (online)	N – 347 (M – 143; F – 204)
**Lewis et al. [31]**	Quest. (retrospective career)	Point	Indirect (online)	N – 2185 (M – 44; F – 2141)
**Puszczałowska-lizis et al. [33]**	Quest. (retrospective career)	Point	-	N – 88 (M – 44; F – 44)
**Ferrante et al. [28]**	Quest. (retrospective career)	Lifetime<br>One-year<br>Six-months<br>Three-months<br>One-month	Indirect (online)	N – 886 (M – 194; F – 692)
**Deckers et al. [20]**	Quest. (retrospective career)<br>Phys. Exam	Lifetime<br>Last month	Direct (each participant)	N – 32 (M – 10; F – 22)
**Cejudo et al. [25]**	Quest. (retrospective 12 mo.)<br>Phys. Exam	One-year	Direct (each participant)	N – 19 (M – 8; F – 11)
**Cejudo et al. [26]**	Quest. (retrospective career)<br>Phys. Exam	One year	Direct (each participant)	N – 43 (M – 15; F – 28)
**Lewis & Baldwin. [21]**	Quest. (retrospective career)	Point	Direct (each participant)	N – 31 (M – 13; F – 18)
**Lewis, Dumbell & Magnoni. [23]**	Quest. (retrospective career)	Point	Indirect (online)	N – 80 (M – 9; F – 71)
**Pilato et al. [29]**	Quest. (retrospective career)	Lifetime	Indirect (email)	N – 73 (M – 2; F – 71)
**Lewis & Kennerley [22]**	Quest. (retrospective career)	Point	Direct (each participant)	N – 50 (F – 50)
**Hobbs et al. [24]**	Quest. (retrospective career)<br>Kinematics	Point	Direct (each participant)	N – 127 (M - 1; F - 126)
**Kraft et al. [32]**	Quest. (retrospective career)<br>Phys. Exams	Point	Direct (each participant)	N – 58 (M – 18; F – 40)

	Clinical exams (retrospective)			
**Kraft et al. [30]**	Quest. (retrospective career)	Point	Indirect (online)<br>Direct	N – 508 (M – 187; F – 321)

(*) number of samples included for each variable was not consistent throughout the study.

Table S8. Detailed data collection tools of included studies.

Reference	Questionnaire tools	Clinical examination tools	Others
**Duarte et al. [27]**	- Self designed questionnaire<br>- Roland Morris Disability questionnaire	-	
**Lewis et al. [31]**	- Self designed questionnaire<br>- McGill Pain Questionnaire<br>- Oswestry Low Back Pain Disability Questionnaire	-	
**Puszczałowska-lizis et al. [33]**	- Self designed questionnaire<br>- Neck Disability Index<br>- Oswestry Low Back Pain Disability Questionnaire	-	
**Ferrante et al. [28]**	- Self designed questionnaire<br>- Standardized Nordic Questionnaires for the analysis of musculoskeletal symptoms<br>- Numeric rating scale<br>- Pain self-efficacy questionnaire	-	
**Deckers et al. [20]**	- Self designed questionnaire<br>- Visual Analog Scale<br>- Oswestry Low Back Pain Disability Questionnaire	- Functional Movement screening tests<br>- Luomajoki's Motor Control screening tool	
**Cejudo et al. [25]**	- Self designed questionnaire	- Tanita-305 body fat analyzer<br>- Sagittal spinal curvatures<br>- ROM-SPORT battery<br>- Trunk muscle endurance	
**Cejudo et al. [26]**	- Self designed questionnaire	- ROM-SPORT I Battery	
**Lewis & Baldwin. [21]**	- Self designed questionnaire<br>- McGill Pain Questionnaire	-	
**Lewis, Dumbell & Magnoni. [23]**	- Self designed questionnaire<br>- McGill Pain Questionnaire<br>- Oswestry Low Back Pain Disability Questionnaire	-	
**Pilato et al. [29]**	- Self designed questionnaire	-	
**Lewis & Kennerley [22]**	- Self designed questionnaire	-	
**Hobbs et al. [24]**	- Self designed questionnaire	- Grip strength<br>- Trunk flexibility	- Images of standing posture<br>- Infra-red motion capture system
**Kraft et al. [32]**	- Self designed questionnaire	- Physical examinations	

	- Visual Analog Scale<br>- Oswestry Low Back Pain Disability Questionnaire	- Magnetic Resonance Imaging	
**Kraft et al. [30]**	- Self designed questionnaire<br>- Visual Analog Scale	-	
