# Supplementary material for: Prevalence of Lower Back Pain and Risk Factors in Equestrians: A Systematic Review
Source: Sports (Basel). 2024 Dec 19;12(12):355. doi: 10.3390/sports12120355 (PMC11679230; doi:10.3390/sports12120355)
Supplement: Supplementary file 1 [file sports-12-00355-s001.zip › Supplementary Materials SII.pdf]

3. Risk of bias and quality assessment OSQE tool

Table S9. Detailed information of OSQE tool – cross-sectional studies, with comments and explanation.

Study	Duarte et al. [27]		Lewis et al. [31]	
	Stars	Comments	Stars	Comments
**Representative**				
Validity	*		*	
Internal validity	1	The study includes a sample of Portuguese equestrian riders, which is relevant to the research question. The sample appears to be diverse and representative of the equestrian population in Portugal	1	The article provides a clear description of the study population, including the selection criteria for participants, which indicates that the sample is representative of the target population of horse riders over thirty-five years old in the UK.
External validity	1		1	
Selection process	1		1	
Reasons refusing	1	No data on refusal.	1	No data on refusal.
**Independent variable**				
Assessment valid	*	The exposure variables related to lower back pain are well-defined and measured using validated questionnaires, ensuring the reliability of the data collected	*	The exposure variables related to riding habits and frequency are well-defined and measured using appropriate and validated tools, ensuring the reliability of the data collected.
Presence optimal	*	Presence of years of sport and workload optimal.	*	Presence of years of sport and workload optimal.
**Dependent variable**				
Assessment	*	The outcome measures for lower back pain prevalence are clearly described and validated, demonstrating that the study effectively captures the intended outcomes	*	The outcome measures concerning pain prevalence are clearly described and validated, demonstrating that the study effectively captures the intended outcomes related to pain experienced by the participants.
**Other**				
Conflict of interest	*	The authors declare no conflicts of interest.	*	The authors declare no conflicts of interest.
**Comparability**				
Confounders control	*	The study identifies potential confounders related to lower back pain and describes the methods used to control for them in the analysis, enhancing the validity of the findings. (logistic, regression)		The statistical analysis of the study is basic and does not control for confounders of interest.
Following a protocol		While the article outlines the statistical methods used for analysis, it does not clearly indicate whether these analyses were pre-registered or determined prior to data collection		While the article outlines the statistical methods used for analysis, it does not clearly indicate whether these analyses were pre-registered or determined prior to data collection
**Optional**				
Missing data	*	No missing data	*	No missing data
Effect modifiers	*	Yes	*	Yes
Systematic review	*	Article mentions sample size and justifies sample size and power analysis.		The article mentions the sample size but does not provide a detailed justification or power analysis to support the adequacy of the sample size.
**Reporting**				
Stating objectives	1	Investigate the prevalence of LBP in Portuguese equestrian athletes and to gain insight into the primary factors or possible causes leading to LBP in this population	1	Investigate the prevalence, location, and severity of pain in riders over the age of 35 years. It also aims to discover factors that affect the pain that riders experience and the pain management techniques used by riders over the age of 35 years
Background provided	1	Yes	1	Yes
Background stratified	1	Yes	1	Yes
Description statistics	1	Yes	1	Yes
Sum of stars - 10	9	No veto ǀMax. sum of stars - 10	7	No veto ǀMax. sum of stars - 10

Table S9 (continuation). Detailed information of OSQE tool – cross-sectional studies, with comments and explanation.

Study	Puszczałowska-lizis et al. [33]		Ferrante et al. [28]	
	Stars	Comments	Stars	Comments
**Representative**				
Validity			*	
Internal validity	1	In- and external validity not optimal (Participants do not represent [age] targeted population in title and objectives). Selection process not transparent. Sample not representative.	1	The study includes a large sample of competitive equestrian athletes from the Italian Equestrian Sport Federation, which enhances the representativeness of the findings for this population.
External validity			1	
Selection process			1	
Reasons refusing	1	No data on refusal.	1	No data on refusal.
**Independent variable**				
Assessment valid	*	The exposure variables related to horse riding practices are well-defined and measured using appropriate methods, ensuring the reliability of the data collected.	*	The exposure variables related to equestrian activities and riding practices are well-defined and measured using appropriate survey methods, ensuring the reliability of the data collected.
Presence optimal	*	Yes	*	Yes
**Dependent variable**				
Assessment	*	The outcome measures for back pain are clearly described and validated, demonstrating that the study effectively captures the intended outcomes	*	The outcome measures for low back pain are clearly described and validated, demonstrating that the study effectively captures the intended outcomes.
**Other**				
Conflict of interest		Authors did not disclose.	*	The authors declare no conflicts of interest.
**Comparability**				
Confounders control		The statistical analysis of the study does not control for confounders of interest.	*	The study identifies potential confounders related to low back pain and describes the methods used to control for them in the analysis, enhancing the validity of the findings.
Following a protocol		While the article outlines the statistical methods used for analysis, it does not clearly indicate whether these analyses were pre-registered or determined prior to data collection		While the article outlines the statistical methods used for analysis, it does not clearly indicate whether these analyses were pre-registered or determined prior to data collection
**Optional**				
Missing data	*	No missing data	*	No missing data
Effect modifiers	*	Yes	*	Yes
Systematic review		The article mentions the sample size but does not provide a detailed justification or power analysis to support the adequacy of the sample size.	*	The article mentions the sample size and provides a detailed justification or power analysis to support the adequacy of the sample size.
**Reporting**				
Stating objectives	1	Analyze the incidence of back pain in people who practice amateur horse riding	1	Investigate, through a self-reported questionnaire, the prevalence of LBP among Italian equestrian athletes; which disciplines in equestrian sports are associated with a higher prevalence of LBP; how the training and competition levels affect the prevalence.
Background provided	1	Yes	1	Yes
Background stratified	1	Yes	1	Yes
Description statistics	1	Yes	1	Yes
Sum of stars - 10	5	No veto │Max. sum of stars - 10	9	No veto │Max. sum of stars - 10

Table S9 (continuation). Detailed information of OSQE tool – cross-sectional studies, with comments and explanation.

Study	Cejudo et al. [25]		Cejudo et al. [26]	
	Stars	Comments	Stars	Comments
**Representative**				
Validity	*		*	
Internal validity	1	The study includes a sample of child equestrian athletes, which is relevant to the research question. The sample appears to be diverse and representative	1	The study includes a sample of child equestrian athletes, which is relevant to the research question. The sample appears to be diverse and representative
External validity	1		1	
Selection process	1		1	
Reasons refusing	1	No data on refusal.	1	No data on refusal.
**Independent variable**				
Assessment valid	*	The exposure variables related to trunk lateral flexor endurance and body fat are well-defined and measured using appropriate methods, ensuring the reliability of the data collected	*	The exposure variables related to asymmetry and range of motion of lower limb muscles are well-defined and measured using appropriate methods, ensuring the reliability of the data collected
Presence optimal	*	Yes	*	Yes
**Dependent variable**				
Assessment	*	The outcome measures for back pain are clearly described and validated, demonstrating that the study effectively captures the intended outcomes.	*	The outcome measures for back pain are clearly described and validated, demonstrating that the study effectively captures the intended outcomes.
**Other**				
Conflict of interest	*	The authors declare no conflicts of interest.	*	The authors declare no conflicts of interest.
**Comparability**				
Confounders control	*	The study identifies potential confounders related to back pain and describes the methods used to control for them in the analysis, enhancing the validity of the findings. (logistic, linear regression).	*	The study identifies potential confounders related to back pain and describes the methods used to control for them in the analysis, enhancing the validity of the findings. (logistic, linear regression).
Following a protocol		While the article outlines the statistical methods used for analysis, it does not clearly indicate whether these analyses were pre-registered or determined prior to data collection		While the article outlines the statistical methods used for analysis, it does not clearly indicate whether these analyses were pre-registered or determined prior to data collection
**Optional**				
Missing data	*	No missing data	*	No missing data
Effect modifiers	*	Yes	*	Yes
Systematic review		While the article mentions the sample size, it does not provide a detailed justification to support the adequacy of this sample size. It reports on power analysis using Gpower.		While the article mentions the sample size, it does not provide a detailed justification to support the adequacy of this sample size. It reports on power analysis using Gpower.
**Reporting**				
Stating objectives	1	Were (I) to analyze the relationship between lower limb ROM (tightness and asymmetry) and LBP and (II) to determine the reference values for lower limb ROM indicating high risk of LBP	1	Determine whether anthropometric, range of motion (ROM), core endurance and sagittal spinal morphotype measures are risk factors for LBP and to establish a diagnostic cutoff value for those factors associated with LBP
Background provided	1	Yes	1	Yes
Background stratified	1	Yes	1	Yes
Description statistics	1	Yes	1	Yes
Sum of stars - 10	8	No veto ǀMax. sum of stars - 10	8	No veto ǀMax. sum of stars - 10

Supplementary Materials SII

Table S9 (continuation). Detailed information of OSQE tool – cross-sectional studies, with comments and explanation.

Study	Deckers et al. [20]		Lewis & Baldwin [21]	
	Stars	Comments	Stars	Comments
**Representative**				
Validity			*	
Internal validity	1	The study includes a sample of horse riders, which is relevant to the research question.	1	The study includes a substantial sample of international event riders competing at a recognized event (Hartpury International Horse Trials), which enhances the representativeness of the findings for this population.
External validity		Sample not representative of sample described in title or aims of the study.	1	
Selection process	1	Selection process transparent.	1	
Reasons refusing	1	No data on refusal.	1	No data on refusal.
**Independent variable**				
Assessment valid	*	The exposure variables related to sport-specific and functional characteristics of back pain are well-defined and measured using appropriate methods, ensuring the reliability of the data collected		Merely descriptive
Presence optimal	*	Yes		Merely descriptive
**Dependent variable**				
Assessment	*	The outcome measures for back pain are clearly described and validated, demonstrating that the study effectively captures the intended outcomes	*	The outcome measures for pain prevalence and its perceived impact on performance are clearly described and validated, demonstrating that the study effectively captures the intended outcomes.
**Other**				
Conflict of interest	*	The authors declare no conflicts of interest.		Authors did not disclose.
**Comparability**				
Confounders control		The statistical analysis of the study does not control for confounders of interest.		The statistical analysis of the study does not control for confounders of interest.
Following a protocol		While the article outlines the statistical methods used for analysis, it does not clearly indicate whether these analyses were pre-registered or determined prior to data collection		While the article outlines the statistical methods used for analysis, it does not clearly indicate whether these analyses were pre-registered or determined prior to data collection
**Optional**				
Missing data	*	No missing data.	*	No missing data.
Effect modifiers	*	Yes	*	Yes
Systematic review		While the article mentions the sample size, it does not provide a detailed justification or power analysis to support the adequacy of this sample size.		The article mentions the sample size (31 questionnaires completed) but does not provide a detailed justification or power analysis to support the adequacy of this sample size.
**Reporting**				
Stating objectives	1	Explore sport-specific and functional characteristics of BP in horse riders.	1	Was to investigate the prevalence of riders at the International CCI*, CCI** and CIC*** levels in eventing competing with pain, the location of their pain, factors affecting their pain and whether they perceived this pain to have an effect on their performance.
Background provided	1	Yes	1	Yes
Background stratified	1	Yes	1	Yes
Description statistics	1	Yes	1	Yes
Sum of stars - 10	6	No veto ǀMax. sum of stars - 10	4	No veto ǀMax. sum of stars - 10

Table S9 (continuation). Detailed information of OSQE tool – cross-sectional studies, with comments and explanation.

Study	Lewis, Dumbell & Magnoni [23]		Lewis & Kennerley [22]	
	Stars	Comments	Stars	Comments
**Representative**				
Validity	*			
Internal validity	1	The study includes a sample of competitive showjumping equestrian athletes, which is relevant to the research question and enhances the representativeness of the findings for this specific population.		In- and external validity not optimal (Participants [sex] do not represent targeted population in title and objectives). Sample not representative of population of interest. Selection process not transparent enough.
External validity	1			
Selection process	1			
Reasons refusing	1	No data on refusal.	1	No data on refusal.
**Independent variable**				
Assessment valid	*	The exposure variables related to pain experienced by the athletes are well-defined, and the study employs a structured questionnaire to gather data, ensuring the reliability of the information collected.		The exposure variables related to pain experienced by the riders are well-defined, and the study employs a structured questionnaire to gather data, ensuring the reliability of the information collected.
Presence optimal		Not controlled		Not possible to assess
**Dependent variable**				
Assessment	*	The outcome measures for pain prevalence and its perceived impact on performance are clearly described and validated, demonstrating that the study effectively captures the intended outcomes.	*	The outcome measures for pain prevalence and its perceived impact on performance are clearly described and validated, demonstrating that the study effectively captures the intended outcomes.
**Other**				
Conflict of interest		Authors did not disclose.		Authors did not disclose.
**Comparability**				
Confounders control		The statistical analysis of the study does not control for confounders of interest.		The statistical analysis of the study does not control for confounders of interest.
Following a protocol		While the article outlines the statistical methods used for analysis, it does not clearly indicate whether these analyses were pre-registered or determined prior to data collection		While the article outlines the statistical methods used for analysis, it does not clearly indicate whether these analyses were pre-registered or determined prior to data collection
**Optional**				
Missing data	*	No missing data.	*	No missing data.
Effect modifiers	*	Yes	*	Yes
Systematic review		The article mentions the sample size but does not provide a detailed justification or power analysis to support the adequacy of this sample size.		The article mentions the sample size but does not provide a detailed justification or power analysis to support the adequacy of this sample size.
**Reporting**				
Stating objectives	1	Investigate the prevalence of competitive showjumping athletes who experience pain, the location of their pain, factors affecting their pain and whether they perceive this pain to effect on their riding performance.	1	Investigate the prevalence of riders at the elite level competing with pain and whether they perceived this pain to have a negative effect on their performance
Background provided	1	Yes	1	Yes
Background stratified	1	Yes	1	Yes
Description statistics	1	Yes	1	Yes
Sum of stars - 10	5	No veto ǀMax. sum of stars - 10	3	No veto ǀMax. sum of stars - 10

Table S9 (continuation). Detailed information of OSQE tool – cross-sectional studies, with comments and explanation.

Study	Pilato et al. [29]		Hobbs et al. [24]	
	Stars	Comments	Stars	Comments
**Representative**				
Validity	*			
Internal validity	1	The study includes a sample of collegiate equestrian athletes, which is relevant to the research question and enhances the representativeness of the findings for this specific population.		In- and external validity not optimal (Participants do not represent targeted population in title and objectives). Sample not representative of population of interest. Selection process not transparent enough.
External validity	1			
Selection process	1			
Reasons refusing	1	No data on refusal.		Sample size varies throughout the study, no explanation provided for this fact, impossible to know if reason for refusal have effects on data representativeness.
**Independent variable**				
Assessment valid	*	The exposure variables related to pain experienced by the athletes are well-defined, and the study employs a structured questionnaire to gather data, ensuring the reliability of the information collected.	*	The exposure variables related to posture, flexibility, and grip strength are well-defined and measured using appropriate methods, ensuring the reliability of the information collected.
Presence optimal	*	Yes	*	Yes
**Dependent variable**				
Assessment	*	The outcome measures for injury history, including spinal injuries, are clearly described and validated, demonstrating that the study effectively captures the intended outcomes.	*	The outcome measures for injury history, including spinal injuries, are clearly described and validated, demonstrating that the study effectively captures the intended outcomes.
**Other**				
Conflict of interest		Authors did not disclose.		Authors did not disclose.
**Comparability**				
Confounders control		The statistical analysis of the study does not control for confounders of interest.	*	The study identifies potential confounders related to posture and strength and describes the methods used to control for them in the analysis, enhancing the validity of the findings (ANOVA).
Following a protocol		While the article outlines the statistical methods used for analysis, it does not clearly indicate whether these analyses were pre-registered or determined prior to data collection		While the article outlines the statistical methods used for analysis, it does not clearly indicate whether these analyses were pre-registered or determined prior to data collection
**Optional**				
Missing data	*	No missing data.		The n of samples included for each variable was not consistent throughout the study.
Effect modifiers*	-	Not applicable (descriptive).	-	Not applicable (differences not association).
Systematic review		The article mentions the sample size but does not provide a detailed justification or power analysis to support the adequacy of this sample size.		The article mentions the sample size but does not provide a detailed justification or power analysis to support the adequacy of this sample size.
**Reporting**				
Stating objectives	1	Analysis is to describe the demographics of collegiate equestrian athletes, their conditioning patterns, their history of pain medication usage and their incidence of injury. Part I includes the demographic data, conditioning patterns, history of pain medication and incidence of injury to the spine. Part II details the incidence of injury for the upper and lower extremity and the head.	1	Determine whether anatomical asymmetry (leg length, pelvis and shoulder height), functional asymmetry (trunk lateral bending and axial rotation range of motion (ROM) during sitting) and dynamical asymmetry (grip strength) were prevalent in a larger population of riders and to determine whether typical traits exist due to riding.
Background provided	1	Yes	1	Yes
Background stratified	1	Yes	1	Yes
Description statistics	1	Yes	1	Yes
Sum of stars – 9*	5	No veto ǀMax. sum of stars - 9	4	No veto ǀMax. sum of stars - 9

Table S9 (continuation). Detailed information of OSQE tool – cross-sectional studies, with comments and explanation.

Study	Kraft et al. [32]		Kraft et al. [30]	
	Stars	Comments	Stars	Comments
**Representative**				
Validity	*		*	
Internal validity	1	The study includes a sample of elite horseback riders, which is relevant to the research question and enhances the representativeness of the findings for this specific population.	1	The study includes a sample of competitive horseback riders from various disciplines, which enhances the representativeness of the findings for this specific population.
External validity	1		1	
Selection process	1		1	
Reasons refusing	1	No data on refusal.	1	No data on refusal.
**Independent variable**				
Assessment valid	*	The exposure variables related to riding discipline, body mass index, and trunk/leg-length coefficient are well-defined and measured using appropriate methods, ensuring the reliability of the information collected.	*	The exposure variables related to riding discipline and intensity are well-defined and measured using appropriate methods, ensuring the reliability of the information collected.
Presence optimal	*	Yes	*	Yes
**Dependent variable**				
Assessment	*	The outcome measures for MRI findings and back pain are clearly described and validated, demonstrating that the study effectively captures the intended outcomes	*	The outcome measures for the incidence of back pain are clearly described and validated, demonstrating that the study effectively captures the intended outcomes.
**Other**				
Conflict of interest	*	The authors declare no conflicts of interest.		Authors did not disclose.
**Comparability**				
Confounders control		The statistical analysis of the study does not control for confounders of interest.		The statistical analysis of the study does not control for confounders of interest.
Following a protocol		While the article outlines the statistical methods used for analysis, it does not clearly indicate whether these analyses were pre-registered or determined prior to data collection		While the article outlines the statistical methods used for analysis, it does not clearly indicate whether these analyses were pre-registered or determined prior to data collection
**Optional**				
Missing data	*	No missing data	*	No missing data
Effect modifiers*	-	Not applicable (mostly descriptive)	*	Yes
Systematic review		While the article mentions the sample size, it does not provide a detailed justification to support the adequacy of this sample size. It reports on power analysis using Gpower.		While the article mentions the sample size, it does not provide a detailed justification to support the adequacy of this sample size. It reports on power analysis using Gpower.
**Reporting**				
Stating objectives	1	Determine whether excessive riding activity accelerates lumbar DD and so leads to lumbar overuse syndromes. Furthermore, we wanted to analyze whether the development of LBP and DDD in the lumbar spine of competitive horseback riders is associated with the riding discipline, body mass index (BMI [kg/m2]), and the trunk/ leg-length coefficient.	1	Assess whether an equestrian discipline leads to increased back pain and to what extent the intensity of equestrian sport is a predisposing factor for the development of this pain. In addition to determining the incidence of back pain in riders, we also wanted to assess whether riding leads to a change in the intensity of pain and whether there is an equestrian discipline that has a positive effect on the symptoms.
Background provided	1	Yes	1	Yes
Background stratified	1	Yes	1	Yes
Description statistics	1	Yes	1	Yes
Sum of stars	7	No veto ǀMax. sum of stars - 9	6	No veto ǀMax. sum of stars - 10

Table S10. Quality score of all included studies and quality taxonomy

Cross-sectional studies	Total number of stars	Max. sum of stars	% Score	Quality assessment
Duarte et al. [27]	9	10	90	High-quality
Lewis et al. [31]	7	10	70	High-quality
Puszczałowska-lizis et al. [33]	5	10	50	Low-quality
Ferrante et al. [28]	9	10	90	High-quality
Cejudo et al. [25]	8	10	80	High-quality
Cejudo et al. [26]	8	10	80	High-quality
Deckers et al. [20]	6	10	60	Low-quality
Lewis & Baldwin [21]	4	10	40	Low-quality
Lewis, Dumbell & Magnoni [23]	5	10	50	Low-quality
Pilato et al. [29]	5	9	55.6	Low-quality
Lewis &1 Kennerley [22]	3	10	30	Low-quality
Hobbs et al. [24]	4	9	44.4	Low-quality
Kraft et al. [32]	7	9	77.8	High-quality
Kraft et al. [30]	6	10	60	Low-quality

Cut-off value 65%.

4. JBI check list for prevalence studies

Table S11. JBI critical appraisal checklist for included studies reporting prevalence data.

	Duarte et al. [27]	Lewis et al. [31]	Ferrante et al. [28]	Lewis & Baldwin [21]	Lewis, Dumbell & Magnoni [23]	Lewis & Kennerley [22]	Hobbs et al. [24]	Kraft et al. [32]	Kraft et al. [30]
1- Was the sample frame appropriate to address the target population?	Y	Y	Y	Y	Y	**N**	Y	Y	Y
2 - Were study participants sampled in an appropriate way?	Y	Y	Y	**U**	Y	**N**	Y	Y	Y
3 - Was the sample size adequate?	Y	**U**	Y	**U**	**U**	**U**	**U**	**U**	Y
4 - Were the study subjects and the setting described in detail?	Y	**U**	Y	**U**	**U**	**U**	**U**	**U**	**U**
5 - Was the data analysis conducted with sufficient coverage of the identified sample?	Y	Y	Y	**N**	Y	**N**	Y	Y	Y
6 - Were valid methods used for the identification of the condition?	Y	Y	Y	Y	Y	Y	**U**	Y	Y
7 - Was the condition measured in a standard, reliable way for all participants?	NA	NA	NA	NA	NA	NA	NA	NA	NA
8 - Was there appropriate statistical analysis?	Y	**U***	**U***	**U***	**U***	**U***	**U***	**U***	**U***
9 - Was the response rate adequate, and if not, was the low response rate managed appropriately?	Y	Y	Y	**N**	Y	Y	Y	Y	Y
Overall appraisal	I	I	I	**E**	I	**E**	I	I	I
Real sample size -precision of the results (%) **	-	2,5	-	**>15**	**13**	**12**	8	9	5
Percentage score (Max. "yes" points - 8) ***	100	62,5	87,5	**-**	62,5	**-**	50	62,5	75
Prevalence study quality assessment	HQ	LQ	HQ	**-**	LQ	**-**	LQ	LQ	HQ

Y – yes; N – No; U – Unclear; NA – Not Applicable; I – Include; E – Exclude; HQ – High-quality; LQ - Low-quality

* For studies that did not present confidence intervals in LBP prevalence, these were calculated by the review team and are presented in table 18. ** For studies that did not justify sample size the SCALEX SP calculator [18] was used, to calculate the precision of the results. A level of confidence of 95% was used for all studies, and loss was considered when authors provided N of non-response or non-completion. Expected prevalence was filled out with the prevalence results of each particular study.

*** Percentage score of all prevalence studies quality to determine quality taxonomy, cut-off value 65%.

Table S12. Confidence intervals of LBP prevalence.

	Prevalence	N total/ N LBP	Lower 95% CL	Prevalence %	Upper 95% CL
Duarte et al. [27]	One-year	347/214	56,5	61,7	66,6
Lewis et al. [31]	Point	2185/969	42,3	44,3	46,4
Ferrante et al. [28]	Lifetime<br>One-year<br>CLBP	886/812<br>886/658<br>886/212	89,6<br>71,3<br>21,2	91,6<br>74,3<br>23,9	93,3<br>77<br>26,8
Lewis & Baldwin [21]	Point	31/16	34,8	51,6	68
Lewis, Dumbell & Magnoni [23]	Point	80/29	26,6	36,2	47,2
Lewis & Kennerley [22]	Point	50/28	42,3	56	68,8
Hobbs et al. [24]	Point	122/34	20,7	27,9	36,4
Kraft et al. [32]	Point	58/51	77,1	87,9	94
Kraft et al. [30]	Point	508/298	54,3	58,7	62,9

Calculated with Epitools – Confidence limits for a sample proportion [19], Confidence level 0.95, Wilson method. Calculated by the review team for all studies that did not provide confidence intervals.
