# Supplementary material for: Prevalence of Lower Back Pain and Risk Factors in Equestrians: A Systematic Review
Source: Sports (Basel). 2024 Dec 19;12(12):355. doi: 10.3390/sports12120355 (PMC11679230; doi:10.3390/sports12120355)
Supplement: Supplementary file 1 [file sports-12-00355-s001.zip › Supplementary Materials SIII.pdf]

Supplementary Materials SIII

Table S13. Population characteristics (demographic and anthropometric) that do not pose a risk or do not contribute to pain.

Anatomic location of pain	Variable	Timeframe/ Details	Not risk factors (no correlation)	Not contributing factors
All body	Prevalence	Point	Age (p=0.114) [21]	
	Intensity		Age (p=0.885) [21]	
			Previous injury (perceived pain) (p=0.781) [21]	
Back	Prevalence	Lifetime		Sex [20]
				Age [20]
	Frequency		Sex (p>0.46) [30]	
	Intensity			Sex [20]
				Age [20]
	Disability			Sex [20]
				Age [20]
Lower back	Prevalence	Lifetime	Sex (p>0,409) [28]	BMI [28]
			Height (p>0,884) [28]	
			Practicing other sports (p>0,6) [28]	
		Point	Incipient disk degeneration (p=0.73) [32]	
		One year	Sex (p=0.243) [27]	
			Sex (p>0,293) [28]	
			Practicing other sports (p=0.210) [27]	
			Practicing other sports (p>0,052) [28]	
			Age (p=0.061) [27]	
			Age (p=0.702) [25]	
			Age (p=0.840) [26]	
			BMI (p=0.178) [27]	
			BMI (p=0.457) [25]	
			BMI (p=0.615) [26]	
			BF% (p=0.626) [26]	
			Height (p>0,839) [28]	
			Height (p=0.282) [25]	
			Height (p=0.881) [26]	
			Weight (p>0,962) [28]	
			Weight (p=0.934) [25]	
			Weight (p=0.775) [26]	
			Asymmetry of ROM in dominant and non-dominant limb (HE, HAD-HF, HAB, HIR, HF-KF) (p<0.04) [25]	
			Asymmetry of ROM in dominant and non-dominant limb (HE, HAB, HIR HAB-HF) (p<0.017) [26]	
			Asymmetry of trunk muscle endurance in dominant and non-dominant limb (ISBE) (p=0.024) [25]	
		Chronic	Sex (p>0,612) [28]	Weight [28]
			Age (p>0,750) [28]	
	Disability	Scores	Sex (p=0.304) [27]	
			Age class (p=0.309) [27]	
			BMI category (p=0.065) [27]	
		Dysfunctional/ Functional	Sex (p=0.171) [27]	
			Practicing other sports (p=0.499) [27]	
			BMI (p=0.075) [27]	
	Disk degeneration	DDD	BMI category (p>0.79) [32]	
			Trunk/ Leg-length coefficient (p>0.73) [32]	

BMI – Body mass index; BF% - Body fat percentage; ROM – Range of motion; HE – hip extension test (iliopsoas); HAD-HF – Hip adduction with hip flexed test (piriformis); HAB – Hip abduction with hip neutral test (adductors); HIR – Hip internal rotation test (external rotators); HF-KF – Hip flexion with knee flexed test (gluteus maximus); ISBE – Isometric side bridge endurance (trunk lateral flexors); HAB-HF – Hip abduction with flexed hip (monoarticular adductors); DDD – Degenerative disk disease;

Table S14. Exposure characteristics (related with Equestrianism) that do not pose a risk or do not contribute to pain.

Anatomic location of pain	Variable	Timeframe/ Details	Not risk factors (no correlation)	Not contributing factors
**All body**	Prevalence	Point		Injuries resulting from falls – 57% [22]
**Back**	Prevalence	Lifetime	Level of competition (Professionals>amateurs) ($p>0.05$) [20]	Years riding [20]
			Equestrian discipline ($p>0.05$) [20]	Workload (H/day) [20]
		Point		Years riding (riders w/ postural defects) [24]
				Equestrian discipline [30]
				Performance classes [30]
				Workload (h/ week) [30]
				Jumping (86.3 % did not affect BP or improved complaints) [30]
	Frequency			Workload (h/week) [30]
				Equestrian discipline [30]
	Intensity		Level of competition (Professionals>amateurs) ($p>0.05$) [20]	Years riding [20]
				Workload (H/day) [20]
				Equestrian discipline [30]
			Equestrian discipline ($p>0.05$) [20]	Workload (h/week) [30]
	Disability		Level of competition (Professionals>amateurs) ($p>0.05$) [20]<br>Equestrian discipline ($p>0.05$) [20]	Years riding [20]<br>Workload (H/day) [20]
**Lower back**	Prevalence	Lifetime	Workload ($p>0,567$) [28]	Sport license [28]
				Years riding [28]
		Point		Workload h/week [32]
		One year	Equestrian sports being a profession vs hobby ($p=0.087$) [27]	
			Equestrian discipline ($p=0.59$) [27]	
			Rider warming up before riding ($p=0.151$) [27]	
			Years riding ($p=0.245$) [27]	
			Years riding ($p=0.557$) [25]	
			Years riding ($p=0.604$) [26]	
			Workload H/Week ($p>0,491$) [28]	
			Workload H/Week ($p=0.089$) [25]	
			Workload H/Week ($p=0.148$) [26]	
			Workload H/Year ($p=0.089$) [25]	
			Workload H/Year ($p=0.148$) [26]	
		Chronic	Sport license ($p>0,178$) [28]	Equestrian discipline [28]
	Intensity			Equestrian discipline [32]
				Workload h/week [32]
	Disability	Scores		Equestrian discipline [32]
		Dysfunctional/ Functional	Workload ($p=0.276$) [27]	
			Performing stable duties ($p=0.077$) [27]	
			Rider warming up before riding ($p=0.948$) [27]	
			Years riding ($p=0.241$) [27]	
	Disk degeneration	T2-Weighted signal intensity	Being an Equestrian athlete ($p>0.46$) [32]	
		DDD	Equestrian discipline ($p>0.15$) [32]	

H/day – hours per day; H/Week – hours per week; H/ Year – hours per year; BP – back pain;

Table S15. Population characteristics (demographic and anthropometric) that pose a risk or contribute to pain.

**Anatomic location of pain**	**Variable**	**Timeframe/ Details**	**Risk factors (correlation)**	**Contributing factors**
**All body**	Prevalence	Point	Age (positive correlation, no *p* values given) [31]	Not practicing other sports (O.R. 1,4) [31]
			Sex (female) (p=0.006) [21]	
**Back**	Prevalence	Lifetime	Lower scores in the in-line lunge test (FMS) (p=0.022) [20]	
			Lower scores in the rocking backwards test (MC) (p=0.014) [20]	
		Point	Age (100% incidence in population 40-45 y.o.) [33]	Age (average age of riders w/ no BP lower than those with frequent symptoms) [30]
			Pain location and sex (p<0.001) [33]	
		One month	Lower scores in the rotary stability test (FMS) (p=0.04) [20]	
			Lower scores in the rocking forwards test (MC) (p=0.02) [20]	
	Intensity		Lower scores in movement control (MC) (p=0.001) [20]	
			Lower scores in functional movement (FMS) (p=0.024) [20]	
	Disability		Lower scores in movement control (MC) (p=0.006) [20]	
			Lower scores in functional movement (FMS) (p<0.001) [20]	
**Lower back**	Prevalence	Lifetime	Younger age (p>0.000) [28]	
			Weight (p>0.003) [28]	
		Point	Pain location and sex (p<0.001) [33]	
		One year	Younger age (p>0.000) [28]	
			BF% (p=0.01) [25]	
			Lower values in trunk muscle endurance (ISBE_ND, ISBE) (p<0.039) [25]	
			Higher values in ROM (HTR) (p=0.043) [25]	
			Lower values in ROM (HAD-HF, KF) (p<0.025) [26]	
	Disability	Scores	BMI (p=0.016) [27]	
		Dysfunctional (as opposed to Functional)	Older age (p=0.022) [27]	

O.R. – Odds ratio; BF% - Body fat percentage; FMS – Functional movement screening tests; MC - Luomajoki's Motor Control screening tool; ISBE -Isometric side bridge endurance (trunk lateral flexors); ISBE_ND – Isometric side bridge endurance in non-dominant side (trunk lateral flexors); ROM – Range of motion; HTR – Hip total rotation (hip rotators); HAD-HF – Hip adduction with flexed hip (abductors); KF – Flexion of knee (quadriceps); y.o. – years old.

Table S16. Exposure characteristics (related with Equestrianism) that pose a risk or contribute to pain.

Anatomic location of pain	Variable	Timeframe/ Details	Risk factors (correlation)	Contributing factors
All body	Prevalence	Point	Years riding (p=0.004) [21]	Saddle – 62% [22]
				Nº of horses ridden – 22% [22]
				Horse's movement – 14% [22]
				Cold weather – 2% [22]
				Years riding, riding for <11 y. leads to < O.R. [31]
				Level of competition (O.R. – leisure 5,46 < amateur 6,30 < professional 7,22) [31]
				Equestrian discipline (eventing) – 96% [21]
		Chronic		Equestrian discipline (Competitive SJ only 2.2 O.R.> Competitive SJ and others 1.5 O.R. (Chronic pain: acute pain)) [23]
	Intensity			Weather (no further explanation) – 41,3% [31]
				Riden activities – 72.8% [31]
				Stable duties – 27.2% [31]
Back	Prevalence	Point		Competition level (riders w/ postural defects) [24]
				Riding (91.5% developed BP during riding career regardless of riding discipline) [30]
		One month	Level of competition (Professional> Amateur) (p=0.014) [20]	
			Saddle type (Jumping saddle> Dressage saddle> Jumping and Dressage saddle) (p=0.027) [20]	
Lower back	Prevalence	Lifetime	Equestrian discipline (Show jumping, Dressage, country riding, reigning, and Eventing) ($p<0.001$) [28]	
		One year	Workload over 7h/ week (p=0.045) [27]	
			Equestrian sports being a profession vs hobby (p=0.039) [27]	
			Performing stable duties (p=0.029) [27]	
		Chronic	Workload (5-6 hours) (p>0.017) [28]	
			Workload (13-18 hours) (p>0.027) [28]	
			Workload (>19 hours) (p>0.043) [28]	
	Intensity			Riding – 42,5% [27]
				Cleaning/ grooming horses – 27,1% [27]
				Lunging horses – 26,2% [27]
				"Mucking out" – 55,1% [27]
	Disability	Scores	Equestrian sports being a profession vs hobby (p=0.017) [27]	Performing stable duties (higher values of estimated marginal means, age and BMI fixed at mean values) [27]
		Dysfunctional (as opposed to Functional)	Equestrian sports being a profession vs hobby (p=0.041) [27]	
	Disk degeneration	T2-Weighted signal alterations		Equestrian discipline (Dressage) [32]

O.R. – Odds ratio.
